# MiR-26a promotes apoptosis of porcine granulosa cells by targeting the 3β-hydroxysteroid-Δ24-reductase gene

**DOI:** 10.5713/ajas.19.0173

**Published:** 2019-07-01

**Authors:** Xiaodong Zhang, Qiangqiang Tao, Jinnan Shang, Yiliang Xu, Liang Zhang, Yingchun Ma, Weihua Zhu, Min Yang, Yueyun Ding, Zongjun Yin

**Affiliations:** 1 College of Animal Science and Technology, Anhui Agricultural University, Hefei 230036, China; 2 Key Laboratory of Local Animal Genetic Resources Conservation and Bio-Breeding of Anhui Province, Hefei, 230036, China

**Keywords:** miR-26a, 3β-hydroxysteroid-Δ24-reductase (*DHCR24*), Apoptosis, Granulosa Cells, Pig

## Abstract

**Objective:**

Apoptosis of ovarian granulosa cells (GCs) affects mammalian follicular development and fecundity. This study aimed to explore the regulatory relationship between microRNA-26a (miR-26a) and the 3β-hydroxysteroid-Δ24-reductase gene (*DHCR24*) gene in porcine follicular granular cells (pGCs), and to provide empirical data for the development of methods to improve the reproductive capacity of pigs.

**Methods:**

The pGCs were transfected with miR-26a mimic, miR-26a inhibitor and *DHCR24*-siRNA *in vitro*. The cell apoptosis rate of pGCs was detected by the flow cytometry. The secretion levels of estradiol (E2) and progesterone (P) in pGCs were detected by enzyme-linked immunosorbent assay. Double luciferase validation system was used to detect the binding sites between miR-26a and *DHCR24* 3′-UTR region. Qualitative real-time polymerase chain reaction and Western blotting were used to verify the *DHCR24* mRNA and protein expression in pGCs, respectively, after transfecting with miR-26a mimic and miR-26a inhibitor.

**Results:**

Results showed that enhancement of miR-26a promoted apoptosis, and inhibited E2 and P secretion in pGCs. Meanwhile, inhibition of *DHCR24* also upregulated the Caspase-3 expression, reduced the BCL-2 expression, promoted pGCs apoptosis, and inhibited E2 and P secretion in pGCs. There were the binding sites of miR-26a located within *DHCR24* 3′-UTR. Up-regulation of miR-26a inhibited *DHCR24* mRNA and protein expression in pGCs.

**Conclusion:**

This study demonstrates that miR-26a can promote cell apoptosis and inhibit E2 and P secretion by inhibiting the expression of *DHCR24* in pGCs.

## INTRODUCTION

The ovary is an important reproductive organ in mammals, and ovulation and hormonal regulation are closely related to reproductive performance [1,2]. The estrous cycle of sow is 21 days on average and can be divided into follicular phase and luteal phase based on ovarian morphological changes and hormone secretion [3]. Apoptosis of porcine ovarian granulosa cells (pGCs) is a physiological phenomenon that occurs during the transition from follicular phase to luteal phase in porcine ovaries, and the percentage of apoptotic granulosa cells (GCs) increases significantly as follicular atresia progress. Previous studies have indicated that miRNAs play important roles in the development of GCs. For example, miR-23 and miR-27a could promote apoptosis of human ovarian GCs [4]. MiR-22 could inhibit apoptosis of mouse ovarian GCs by targeting the sirtuin 1 gene [5]. Liu et al [6] reported that the hyaluronan synthase 2 (*HAS2*) gene is a direct target of miR-26b in pGCs, and miR-26b positively regulates pGCs apoptosis via a HAS2-HA-CD44-Caspase-3 pathway by targeting the *HAS2* gene. MiR-26a has the same seed sequence with the miR-26b and has been reported as a tumor suppressor in liver cancer cells [7,8], osteosarcoma cells [9] and papillary thyroid cancer cells [10]. Studies have also shown that miR-26a regulates the osteogenic differentiation of bone marrow mesenchymal stem cells [11], the proliferation of mouse hepatocyte [12], the apoptosis of endothelial cells [13] and the autophagy of swine Sertoli cells [14]. However, the regulation mechanism of miR-26a in ovarian function and follicular development is still rarely reported. Only one research found that miR-26a/b might play a significant role in follicular development by targeting the mothers against decapentaplegic homolog 2 (*SMAD2*) gene [15]. Our previous researches indicated that the 3β-hydroxysteroid-Δ24-reductase (*DHCR24*), which encodes for the 24-dehydrocholesterol reductase protein, was a candidate target gene of miR-26a in porcine ovaries [16,17]. The *DHCR24* gene is the final catalytic enzyme involved in cholesterol synthesis, which catalyzes the reduction of streptavidin to cholesterol [18]. The *DHCR24* gene also may play an important role in stress signaling pathways and apoptosis [19–21]. Therefore, in this study, we studied the effects of abnormal expression of miR-26a and *DHCR24* on apoptosis and hormone secretion of pGCs and elucidated the regulatory relationship between miR-26a and *DHCR24*. The results show that miR-26a promotes apoptosis of pGCs by targeting the 3′UTR of *DHCR24* and regulates the post-transcriptional expression of *DHCR24*. The empirical data reported in this paper can provide genetic information for elucidating the apoptotic mechanism of ovarian GCs.

## MATERIALS AND METHODS

### Ethics statement

Experimental pigs were allowed access to feed and water *ad libitum* under normal condition and were sacrificed humanely to minimize suffering. All experimental procedures and sample collection were approved by the Institutional Animal Care and Use Committee of Anhui Agricultural University, Anhui, China under permit No. 20160523.

### Cell culture

Fresh porcine ovaries were obtained from a commercial slaughter house and transported back to the laboratory within 1 h. The pGCs were collected from porcine ovarian follicles (3 to 6 mm diameter). The cells were seeded into a 60 mm dish and cultured at 37°C and 5% CO_2_ in Dulbecco’s modified eagle medium (DMEM)/F-12 medium (Gibco, Carlsbad, CA, USA) containing 10% fetal bovine serum (FBS) (Gibco, USA), 100 units/mL penicillin, and 100 mg/mL streptomycin (Gibco, USA). The 293T cells were incubated at 37°C and 5% CO_2_ in DMEM containing 10% FBS.

### Oligonucleotide transfection

The pGCs were collected at 48 h after transfection. pGCs were transfected with miR-26a mimic, non-targeting control oligonucleotide (NC mimic), miR-26a inhibitor, non-targeting inhibitor oligonucleotide (NC inhibitor), *DHCR24*-siRNA and NC-siRNA. These oligonucleotide sequences were designed based on the porcine miR-26a mature sequence in the miRBase database (http://www.mirbase.org) and the *DHCR24* sequence in the GenBank database (https://www.ncbi.nlm.nih.gov/genbank) and were synthesized from Ribobio Co. Ltd (Ribobio, Guangzhou, China) (Table 1). Transfection was performed using Lipofectamine 3000 reagent (Invitrogen, Waltham, MA, USA). Briefly, pGCs were seeded in 12-well or 6-well plates at 1 d prior to transfection. When the cells reached 60% to 70% coverage of one well, miRNAs and siRNAs were transfected into the cells at different final concentrations. The final concentrations of miR-26a mimic, NC mimic, *DHCR24*-siRNA, and NC-siRNA were 100 nM. The final concentrations of miR-26a inhibitor and NC-inhibitor were 200 nM. All experiments were performed in triplicate.

### Quantitative real-time polymerase chain reaction

Total RNA was extracted by RNA extraction kit (OMEGA, Norcross, GA, USA) and then reverse transcribed using a TransScript Green miRNA First-Strand cDNA Synthesis SuperMix kit (TransGen Biotech, Beijing, China) for miRNA and a TransScript One-Step DNA Removal and cDNA Synthesis SuperMix kit (TransGen Biotech, China) for mRNAs. Quantitative real-time polymerase chain reaction (qPCR) was performed using SYBR Premix Ex Taq (TaKaRa, Osaka, Japan) and a CFX96 real-time PCR Detection System (Bio-Rad, Hercules, CA, USA), following the manufacturer’s instructions. Relative gene expression values were determined using the 2^−ΔΔCt^ method. The primers used are listed in Table 2. U6 small nuclear RNA and β-actin were used as endogenous internal controls for miRNA and mRNA expression, respectively.

### Western blotting

The pGCs were collected 72 h after transfection, and whole cell lysates were prepared in radio immunoprecipitation assay buffer (50 mM Tris HCl, pH 8, 150 mM NaCl, 1% Nonidet P-40, 0.1% sodium dodecyl sulfate [SDS], 1% Triton X-100, and proteinase inhibitors) (Solarbio, Beijing, China). Sample protein concentrations were determined by the bicinchoninic acid method (Pierce, Shanghai, China). Protein samples were separated by SDS-polyacrylamide gel electrophoresis (PAGE). Briefly, a 10% SDS-PAGE gel was prepared and 20 μg of protein was loaded per sample. After electrophoresis for 1 h, the proteins were transferred to a polyvinylidene fluoride membrane (Millipore, Billerica, MA, USA). The membrane was blocked in 5% non-fat milk and then incubated at 4°C overnight with a diluted (1:500) monoclonal anti-*DHCR24* antibody (Bioss, Beijing, China) or anti-β-actin antibody (as an internal loading control) (Bioss, China), followed by incubation with secondary antibody (1:2,000) for 2 h at room temperature. The specific complexes were visualized using the SuperSignal West Pico chemiluminescent substrate. Densitometric analysis was performed to quantify the signal intensity.

### Apoptosis analysis

After cells were transfected and incubated for 48 h, cells were dissociated with trypsin and resuspended in 500 μL binding buffer containing 5 μL annexin V-fluorescein isothiocyanate and 10 μL propidium iodide (Bestbio, Shanghai, China). The counts of stained cells were determined using a FACSCalibur flow cytometry instrument (BD Biosciences, Franklin Lakes, NJ, USA). All experiments were performed at least three times.

### Luciferase reporter assay

For miR-26a-binding site detection, the GP-miRGLO Dual-luciferase miRNA Target Expression Vector, containing the wild-type *DHCR24* 3′-UTR, was constructed. For *DHCR24*-binding site detection, the GP-miRGLO reporter vector containing the wild-type and mutated miR-26a promoter was constructed. Mutant plasmids were constructed using a MutanBEST Kit (TaKaRa, Japan), according to the manufacturer’s instructions. 293T cell was plated in 12-well plates and transfected with 1.6 μg constructed plasmids and miR-26a mimic or NC mimic. After transfection for 48 h, 293T cell was collected and luciferase activity was measured using the Dual-Luciferase Reporter Assay System (Promega, Madison, WI, USA), according to the manufacturer’s instructions.

### Enzyme-linked immunosorbent assay

After 48 hours transfection, pGC culture medium containing 10% FBS was collected by centrifugation at 2,000×g for 20 min to measure estradiol (E2) and progesterone (P) concentrations using pig E2 and P ELISA kits (Ji Yin Mei, Wuhan, China), respectively, according to manufacturer’s instructions.

### Statistical analyses

Differential analysis was performed using IBM SPSS Statistics v20.0 (SPSS Inc., Chicago, IL, USA). Unpaired two-sided Student’s t-tests and one-way analysis of variance tests were used to evaluate the significance of the statistics. Statistical significance is defined when p values are less than 0.05.

## RESULTS

### MiR-26a promotes apoptosis and decreases E2 and P secretion in porcine ovarian granulosa cells

**Figure 1:**
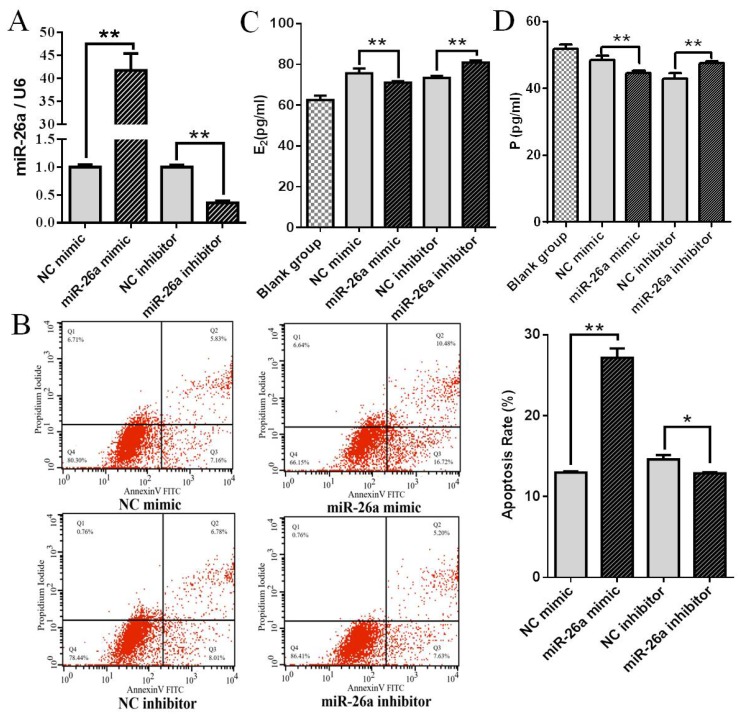
miR-26a regulates apoptosis rate and E2 and P release in pGCs. (A) qPCR validation of miR-26a overexpression and inhibition using mimic and inhibitor. (B) The pGCs transfected with miR-26a mimic or inhibitor were subjected to Annexin V-FITC/PI double staining and flow cytometric analysis. (C, D) The levels of E2 and P were detected by ELISA. The blank group was the E2 or P content in the culture media supplemented with fetal bovine serum. Average results from three independent experiments are shown. E2, estradiol; P, progesterone; pGCs, porcine ovarian granulosa cells; qPCR, qualitative real-time polymerase chain reaction; ELISA, enzyme-linked immunosorbent assay; NC, negative control. * p<0.05, ** p<0.01.

To determine whether miR-26a plays a role in controlling apoptosis in pGCs, miR-26a mimic and miR-26a inhibitor were transfected into cultured pGCs. Apoptosis was evaluated in transfected pGCs using annexin V FITC/PI staining and flow cytometry analysis. Compared with NC mimic and NC inhibitor, the expression of miR-26a was significantly upregulated and downregulated after transfecting with miR-26a mimic and miR-26a inhibitor, respectively (p<0.01) (Figure 1A). Cell apoptosis was significantly higher in pGCs transfected with miR-26a mimic than in pGCs transfected with NC mimic (p<0.01). Likewise, apoptosis in pGCs transfected with miR-26a inhibitor was significantly lower than pGCs transfected with NC inhibitor (p<0.05) (Figure 1B). These results indicate that miR-26a promotes apoptosis and is a proapoptotic factor in pGCs. Meanwhile, miR-26a mimic significantly decreased E2 and P release, and miR-26a inhibitor significantly promoted E2 and P release in cultured pGCs (Figure 1C, 1D).

### Inhibition of *DHCR24* induces apoptosis and decreases E2 and P secretion in porcine ovarian granulosa cells

**Figure 2:**
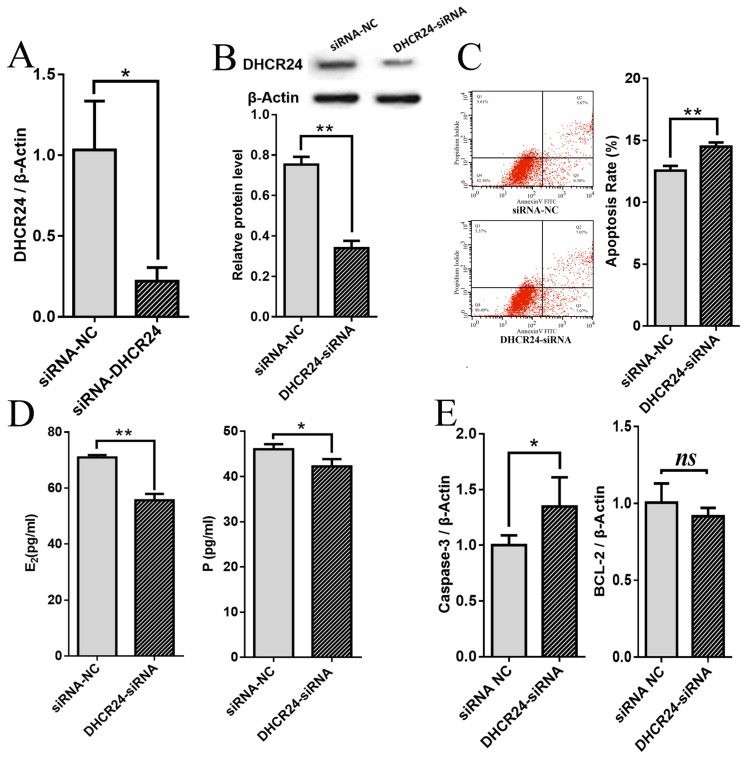
Effects of DHCR24 on the apoptosis rate, E2 and P release and Caspase-3 and BCL-2 mRNA levels in pGCs. qPCR (A) and Western blot (B) analyses of *DHCR24* mRNA and protein expression, respectively, in pGCs with NC-siRNA or *DHCR24*-siRNA. (C) pGCs transfected with *DHCR24*-siRNA or NC-siRNA were subjected to Annexin V-FITC/PI double staining and flow cytometric analysis. Knockdown of *DHCR24* accelerates the apoptosis rate of pGCs. (D) pGCs transfected with *DHCR24*-siRNA or siRNA-NC. The levels of E2 and P were detected by ELISA. (E) qPCR analyses showed that knockdown of *DHCR24* increased the mRNA level of Caspase-3, but did not affect the mRNA level of BCL-2 in pGCs. β-Actin was used as an internal control. Average results from three independent experiments are shown. DHCR24, 3β-hydroxysteroid-Δ24-reductase; E2, estradiol; P, progesterone; pGCs, porcine ovarian granulosa cells; qPCR, qualitative real-time polymerase chain reaction; NC, negative control; ELISA, enzyme-linked immunosorbent assay. * p<0.05, ** p<0.01.

To explore the function of *DHCR24* in apoptosis of pGCs, RNA interference was used to inhibit *DHCR24* expression in pGCs cultured *in vitro*. Successful knockdown of *DHCR24* confirmed by qPCR analysis; compared with pGCs transfected with the NC-siRNA, those transfected with the *DHCR24*-siRNA had significantly lower *DHCR24* mRNA expression (p<0.05) (Figure 2A). Consistent with the mRNA expression, the expression level of *DHCR24* protein was also down-regulated significantly as a result of the specific siRNA treatment (p<0.01) (Figure 2B). Flow cytometry analysis revealed that the rate of apoptosis in the *DHCR24*-siRNA group was significantly higher than in the NC-siRNA group (p<0.01) (Figure 2C). In addition, inhibition of *DHCR24* expression reduces the secretion of E2 and P in pGCs (Figure 2D). Compared with the NC-siRNA group, the expression level of the proapoptotic Caspase-3 gene was increased significantly in the *DHCR24*-siRNA group (p<0.05), while that of the antiapoptotic BCL-2 gene was not changed (Figure 2E). These results indicate that inhibition of *DHCR24* expression enhances apoptosis of pGCs in porcine ovaries.

### DHCR24 is a direct target of miR-26a

**Figure 3:**
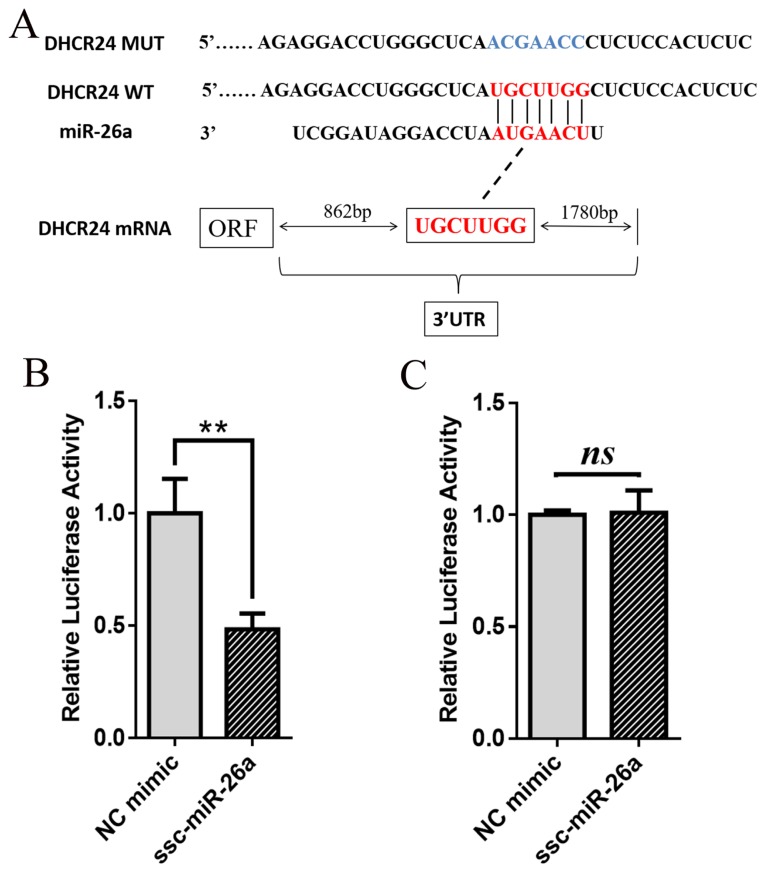
DHCR24 is a direct target of miR-26a. (A) Schematic showing the interactions of miR-26a with wild-type *DHCR24* 3′-UTR (red) and the mutant version (blue). (B and C) Transfection of 293T cells with miR-26a mimic and a dual-luciferase reporter vector containing the wild-type *DHCR24* 3′-UTR (B) or the mutant version (C). Luciferase activity was measured and normalized to that in the NC mimic treated group. Average results from three independent experiments are shown. DHCR24, 3β-hydroxysteroid-Δ24-reductase; NC, negative control. * p<0.05, ** p<0.01.

To determine whether miR-26a is able to regulate *DHCR24* gene expression, the putative miR-26a target sites in the porcine *DHCR24* 3′-UTR were cloned downstream of the luciferase gene in the pmirGLO dual-luciferase reporter vector to generate pmirGLO-*DHCR24*-3′-UTR (Figure 3A). 293T cells were transiently co-transfected with the reporter plasmid and with miR-26a mimic or NC mimic oligos. Overexpression of exogenous miR-26a repressed the activity of the luciferase reporter fused to the *DHCR24* 3′-UTR (p<0.01) (Figure 3B), however, the luciferase activity was not altered significantly when the cells were co-transfected with the miR-26a mimic and a *DHCR24* reporter 3′-UTR construct containing a mutation in the putative miR-26a binding site (Figure 3C). These results indicate that miR-26a can regulate the expression of the porcine *DHCR24* gene by binding to conserved sites in the *DHCR24* 3′-UTR.

### MiR-26a inhibits *DHCR24* expression in porcine ovarian granulosa cells

**Figure 4:**
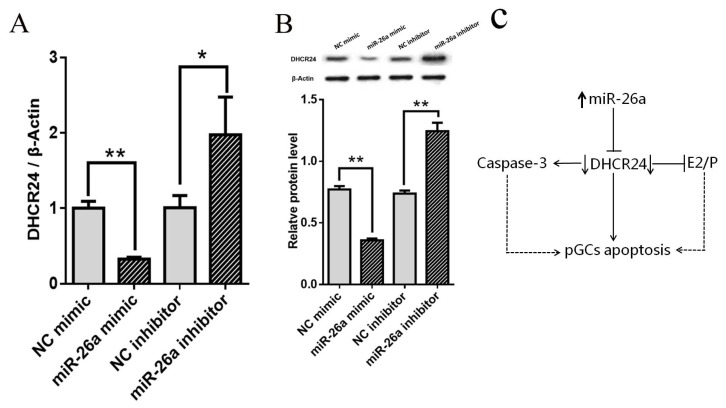
Effects of miR-26a on the expression of DHCR24 in pGCs. (A) NC mimic, miR-26a mimic, NC inhibitor, or miR-26a inhibitor were transfected into pGCs. *DHCR24* mRNA levels were detected by qPCR analysis. (B) NC mimic, miR-26a mimic, NC inhibitor, or miR-26a inhibitor were transfected into pGCs. *DHCR24* protein expression was detected by western blotting. β-Actin was used as an internal control. NC means negative control. Average results from three independent experiments are shown. * p<0.05, ** p<0.01. (C) A model for miR-26a regulates pGCs apoptosis by targeting *DHCR24*. MiR-26a inhibits the expression of *DHCR24* gene, which leads to an increase in expression of the proapoptotic Caspase-3 gene and inhibition in the secretion of E2/P and promotion of apoptosis level in pGCs. DHCR24, 3β-hydroxysteroid-Δ24-reductase; pGCs, porcine ovarian granulosa cells; qPCR, qualitative real-time polymerase chain reaction.

To confirm that miR-26a promotes apoptosis in pGCs by targeting the *DHCR24* gene, the *DHCR24* mRNA and protein levels were measured after transfection of cultured pGCs with miR-26a mimic and miR-26a inhibitor. The qPCR analysis revealed that *DHCR24* mRNA expression was significantly lower in the pGCs transfected with the miR-26a mimic than with the NC mimic (p<0.01) (Figure 4A). Expression of *DHCR24* mRNA in pGCs transfected with miR-26a inhibitor was significantly higher than those transfected with the NC inhibitor (p<0.05) (Figure 4A). Meanwhile, trends in *DHCR24* protein expression were consistent with changes in *DHCR24* mRNA expression (Figure 4B). These results suggest that miR-26a accelerates apoptosis in pGCs by inhibiting the mRNA and protein expression of *DHCR24*.

## DISCUSSION

The mammalian ovary is a dynamic organ. Follicular recruitment, selection and ovulation coordination, and timely development are essential for functional ovaries and fertility [22]. With the apoptosis of ovarian GCs, follicular atresia gradually occurred. Previous studies have shown that apoptosis of ovarian GCs is the direct cause of follicular atresia [23], and once follicles enter the atresia process, it will be irreversible [24].

In recent years, the regulation of miRNAs in mammalian ovarian development has attracted much attention. miRNA can affect apoptosis of ovarian GCs, oocyte development, and hormone secretion, which are closely associated with mammalian reproductive traits [25,26]. MiR-26a shares the same seed sequence as miR-26b, which has also been reported to be involved in the regulation of animal reproduction. MiR-26b can regulate the apoptosis of porcine follicular GCs by targeting the *Smad4* gene [27]; likewise, miR-26a/b can target the *Smad2* gene and regulate bovine follicular development [15]. MiR-26a plays a role in regulating cell proliferation and apoptosis in embryonic stem cells of dairy goats by directly regulating the phosphatase and tensin homolog gene, and miR-26a can indirectly regulate the PI3K/AKT pathway in endometrial epithelium cells [28]. However, studies on miR-26a in pGCs have not been reported. In this study, we found that transfection of miR-26a mimics promoted apoptosis of pGCs, and inhibition of miR-26a expression suppressed apoptosis of pGCs. These results are consistent with the observed functions of miR-26a in other cells. These results suggest that miR-26a is a proapoptotic factor in pGCs.

The *DHCR24* gene encodes for the 24-dehydrocholesterol reductase protein, which is the final catalytic enzyme involved in cholesterol synthesis, which catalyzes the reduction of streptavidin to cholesterol [18]. Previous studies have shown that *DHCR24* plays an important role in stress signaling pathways and apoptosis. Up-regulation of *DHCR24* protein expression can inhibit Caspase-3 initiation during cellular stress responses, thereby acting as an anti-apoptotic agent and displaying neuroprotective effects [29]. Overexpression of *DHCR24* in neurons cells has anti-apoptotic effects and counters oxidative stress by scavenging free radicals [30]. Previous studies also showed that 17-β estradiol can promote *DHCR24* expression and can increase intracellular cholesterol content, which protects neurons cultured *in vitro* [31]; however, this protection disappears after knocking out *DHCR24*, indicating that *DHCR24* may be a sex hormone-mediated regulator of neuroprotection [32]. Meanwhile, the estrogen signaling plays a critical role in the development of the female reproductive system, and the generation of a primordial follicle may be dependent on both estrogen and ER-α signaling pathways [33]. In this study, we found that inhibition of *DHCR24* expression can significantly increase Caspase-3 expression, promote apoptosis, and inhibit estradiol and progesterone secretion in pGCs. Because of *DHCR24* is the final catalytic enzyme involved in cholesterol synthesis, which catalyzes the reduction of 24-dehydrocholesterol to cholesterol, we predicted that inhibition of *DHCR24* could promote apoptosis by inhibiting the secretion of estradiol and progesterone in pGCs. However, the accurate regulation mechanism still needs to be further studied.

MiRNAs are widely involved in the regulation of gene ex pression by destabilizing mRNA transcripts and interfering with post-transcriptional protein translation [34,35]. An established approach to elucidate the function of a miRNA is to identify genes that are predicted to be regulated by the miRNA [36]. In this study, we predicted the binding sites of miR-26a in the 3′-UTR region of *DHCR24* by the RNAbybird software. Although there were two mismatches (G-U) in the seed sequences, they did not affect the results of the double luciferase reporter gene assay. Several previous studies also showed there was the mismatch of G-U in RNA sequence[37,38]. The results indicate that miR-26a can regulate the expression of the porcine *DHCR24* gene by binding to conserved sites in the *DHCR24* 3′-UTR.

In conclusion, our data provide direct evidence that miR-26a can induce apoptosis of pGCs and inhibit the secretion of estrogen and progesterone by inhibiting the expression of *DHCR24* gene (Figure 4C). These findings provide novel insights into the mechanisms underlying apoptosis of GCs, follicular atresia, and development in mammalian ovaries.

## Figures and Tables

**Table 1:** The sequences of oligonucleotide used in this study

Name	Sequence (5′→3′)
mimics NC	F: UUU AGC AUU GAA GGU CAA CGC A
	R: UGC GUU GAC CUU CAA UGC UAA A
miR-26a mimics	F: UUC AAG UAA UCC AGG AUA GGC U
	R: AGC CUA UCC UGG AUU ACU UGA A
Inhibitor NC	CAG UAC UUU UGU GUA GUA CAA
miR-26a inhibitor	AGC CUA UCC UGG AUU ACU UGA A
*DHCR24*-siRNA	CTA CCT GAA GAC AAA CCA A

NC-siRNA was synthesized by Ribobio (Ribobio, Guangzhou, China) and its sequence was kept confidential.

NC, negative control; *DHCR24*, 3β-hydroxysteroid-Δ24-reductase.

**Table 2:** The primers of the mRNAs and miRNAs for qualitative real-time polymerase chain reaction

Primer name	Primer sequences	Tm (°C)	Length (bp)
*DHCR24* F	CAGAAATCCCACCCAGAGAG	58	210
*DHCR24* R	GACAGCCAACAGGCAGATAG		
Bcl-2-F	CTTTGCCGAGATGTCCAGC	60	197
Bcl-2-R	TCCACAGGGCGATGTTGTC		
Caspase-3-F	TAACCCGAGTAAGAATGT	51	160
Caspase-3-R	ATACCAGTTGAGGCAGAC		
β-Actin F	CTCGATCATGAAGTGCGACG	60	114
β-Actin R	GTGATCTCCTTCTGCATCCTGTC		
miR-26a	GCTTCAAGTAATCCAGGATAGGCT		
U6 snRNA	GGCAAGGATGACACGCAAAT		

*DHCR24*, 3β-hydroxysteroid-Δ24-reductase.
